# Environmental enrichment influences novelty reactivity, novelty preference, and anxiety via distinct genetic mechanisms in C57BL/6J and DBA/2J mice

**DOI:** 10.1038/s41598-021-83574-6

**Published:** 2021-02-16

**Authors:** Price E. Dickson, Guy Mittleman

**Affiliations:** 1 Department of Psychology, University of Memphis, 400 Innovation Drive, Memphis, TN 38111 USA; 2 Department of Biomedical Sciences, Joan C. Edwards School of Medicine, Marshall University, 1700 3rd Ave., Huntington, WV 25703 USA; 3 Department of Psychological Science, Ball State University, North Quad (NQ), room 104, Muncie, IN 47306 USA

**Keywords:** Epigenetics and behaviour, Reward

## Abstract

Environmental factors such as stress drive the development of drug addiction in genetically vulnerable individuals; the genes underlying this vulnerability are unknown. One strategy for uncovering these genes is to study the impact of environmental manipulation on high-throughput phenotypes that predict drug use and addiction-like behaviors. In the present study, we assessed the viability of this approach by evaluating the relative effects of environmental enrichment and isolation housing on three high-throughput phenotypes known to predict variation on distinct aspects of intravenous drug self-administration. Prior to behavioral testing, male and female C57BL/6J and DBA/2J mice (BXD founders) were housed in enrichment or isolation for ten weeks beginning at weaning. Enrichment significantly reduced novelty reactivity; this effect was significantly more robust in C57BL/6J mice relative to DBA/2J mice. Enrichment significantly reduced novelty preference; this effect was significantly dependent on novel environment characteristics and was significantly more robust in DBA/2J mice relative to C57BL/6J mice. Enrichment significantly increased anxiety; this effect was not strain-dependent. Collectively, these data indicate that (1) environmental enrichment influences novelty reactivity, novelty preference, and anxiety via distinct genetic mechanisms in mice, and (2) the BXD panel can be used to discover the genetic and epigenetic mechanisms underlying this phenomenon.

## Introduction

Drug addiction is a heritable disease driven by unknown genetic and environmental factors^1^. Intravenous drug self-administration in mice^2^ provides a direct but technically challenging approach for discovery of genetic and environmental factors driving drug use and addiction-like behaviors. A complementary strategy for uncovering these mechanisms is the study of easily quantifiable behavioral phenotypes (e.g. novelty reactivity, novelty preference, and anxiety) that predict drug use and addiction-like behaviors^3–8^. A distinct advantage of using these predictive phenotypes as a proxy for intravenous drug self-administration is that they can be quantified in mice using high throughput, low stress protocols that require no drug exposure. These characteristics allow quantification of multiple predictive phenotypes in the same mouse and same study using identical experimental conditions; this allows direct comparison of genotype, environment, and sex effects across phenotypes. In summary, this strategy provides an easily accessible window onto genes that have a high probability of influencing drug use and addiction-like behaviors.

Epidemiological studies reveal that environmental stress drives the development of drug addiction in genetically vulnerable individuals^9^; the genes underlying this vulnerability are largely unknown. In experimental animals, isolation housing^10^ and environmental enrichment^11^ provide rodent models of high-stress and low-stress environments, respectively^12,13^. Studies in mice and rats using these models reveal that isolation housing potentiates, and environmental enrichment attenuates, both drug use and phenotypes that predict drug use^12–15^. Together with observations of correlated drug use phenotypes and predictive phenotypes^3–8^, these data suggest that the biological mechanisms influencing both drug use and predictive phenotypes are themselves influenced by housing manipulation. Consequently, a strategy of housing manipulation (i.e., enrichment vs. isolation) followed by quantification of predictive phenotypes could be used to identify mouse strains with alleles that confer vulnerability or resistance to addiction following stress exposure. Employing this strategy using a recombinant inbred mouse panel (e.g., BXD^16^, Collaborative Cross^17^) in the context of a systems genetics approach would enable the discovery of the precise genetic mechanisms underlying these strain differences in stress-induced addiction vulnerability.

In the present study, we assessed the effects of housing condition, strain, sex, and the interaction among these factors on three phenotypes that have previously been shown to predict substance use variation in mice, rats, or both: novelty reactivity, novelty preference, and anxiety^3–6^. To index these phenotypes, we used exploration of a novel open field, preference for a novel chamber in a conditioned place preference apparatus, and preference for the closed arms in an elevated zero maze, respectively. We assessed these effects in the two founder strains of the BXD recombinant inbred panel (C57BL/6J and DBA/2J) to determine if a larger systems genetics study using the BXD panel^7,18^ would be feasible. Specifically, our goal was to determine if C57BL/6J alleles and DBA/2J alleles differentially influence the effect of housing manipulation on the assessed predictive phenotypes.

Male and female C57BL/6J and DBA/2J mice were weaned at four weeks and housed in enriched or isolated housing for ten weeks prior to behavioral testing. Enriched mice were housed in large cages with conspecifics, exercise wheels, nesting materials, and toys. Isolated mice were singly housed in standard shoebox cages without enrichment items. To control for variation of environmental variables that were not explicitly manipulated, each isolation-housed mouse was matched to an enrichment-housed littermate; these littermates were housed side-by-side on the same shelf throughout the experiment and tested in the same apparatus. Following the experiment, data were analyzed to assess the effects of the three independent variables on the addiction-predictive behavioral endpoints.

## Materials and methods

### Subjects

Experiments were conducted in The Department of Psychology at The University of Memphis and approved by the Institutional Animal Care and Use Committee at the University of Memphis. Experiments were conducted in accordance with the National Institutes of Health Guidelines for the Care and Use of Laboratory Animals and with the ARRIVE guidelines. Efforts were made to reduce the number of animals used and to minimize animal pain and discomfort.

Male and female C57BL/6J mice (stock number: 000664) and DBA/2J mice (stock number: 000671) were purchased from The Jackson Laboratory (Bar Harbor, ME). A single male and a single female of the same strain were housed together in standard cages on ventilated racks. Offspring from these breeder pairs were weaned at four weeks of age and were used as experimental subjects. Assignment to experimental conditions occurred as follows: Two mice of the same sex were randomly selected from a litter and those mice were randomly assigned to the isolation housing condition or environmental enrichment condition. Mice were housed in these conditions for 10 weeks at which point testing began. Mice remained housed in isolation or environmental enrichment conditions on the days that they were tested apart from the brief time that they were in the testing apparatus. Mice were maintained in a temperature-controlled environment (21 ± 1 °C) on a 12:12 light:dark cycle (lights on at 0800). Mice had free access to food and water throughout the experiment with the exception of the brief time in the testing apparatus.

### Environmental enrichment

Mice in the isolation housing condition were housed individually in clear polycarbonate standard size mouse pens (11.5″ L × 7.5″ W × 5″ H). Aspen wood bedding was used to line the bottom of the pen. No other items were placed in the pen. Mice in the environmental enrichment condition were housed in same-sex groups in clear polycarbonate standard size rat pens (19.5″ L × 10″ W × 8″ H) which were significantly larger than a standard size mouse pen. Three C57BL/6J mice and three DBA/2J mice were housed together in each environmental enrichment pen. In addition to bedding, each environmental enrichment pen contained the following items: both a vertical and a horizontal running wheel for exercise; an opaque PVC tube and one half of a glove box for shelter; three Nestlets for nest building. Isolation housed mice and their enrichment housed littermates were part of the same experimental cohort. Enrichment housed and isolation housed mice from the same cohort were maintained side-by-side on the same shelf in the mouse housing room to ensure they were exposed to the same microenvironment with the exception of experimentally-manipulated housing variables.

### Apparatus

#### Novelty reactivity

Novelty reactivity testing was performed using six standard Med Associates (St. Albans, VT) mouse open field arenas (ENV-510) with dimensions of 10.75″ L × 10.75″ W × 8″ H. Each arena was constructed of clear acrylic walls and a white opaque base. A white opaque cover was placed over the top of each arena during testing to prevent mice from jumping out of the apparatus. Integrated IR controllers (ENV-520) in combination with associated hardware and software allowed for automated data collection. Each open field apparatus was housed within a sound attenuating cubicle (ENV-022V) containing a ventilation fan to reduce ambient noise.

#### Novelty preference

Novelty preference testing was performed using six standard Med Associates conditioned place preference apparatus for mice (MED-CPP-MS) with overall interior dimensions of 18.4″ L × 5″ W × 5″ H. Each apparatus consisted of a center compartment (4.6″ L × 5″ W × 5″ H) with a neutral gray finish and a smooth PVC floor, (2) a left compartment (6.9″ L × 5″ W × 5″ H) with white walls and a stainless steel wire mesh floor, and (3) a right compartment (6.9″ L × 5″ W × 5″ H) with black walls and a stainless-steel bar floor. The center compartment was connected to the left and right compartments via manually operated guillotine doors. A hinged clear polycarbonate lid over each compartment allowed for loading and unloading of mice and prevented escape from the apparatus during testing. Activity level and percentage of time in the different compartments were collected by means of an integrated array of infrared detectors and associated hardware and software.

#### Anxiety

Anxiety testing was performed using three standard AccuScan Instruments Inc. (Columbus, OH) elevated zero mazes separated by opaque partitions. Each maze was elevated 43″ from the floor and consisted of a black circular platform (2″ W with 16″ outer diameter) evenly divided into 2 closed and 2 open quadrants. The closed quadrants had clear acrylic walls (11″ H) on the inside and outside of the maze platform. The open quadrants did not have walls. During testing, each maze was illuminated by a 15 W red light bulb suspended 49″ above the maze platform. Activity level and percentage of time in the open and closed quadrants of each maze were collected using integrated IR detectors and associated hardware and software.

### Experimental procedures and dependent variables

Mice were tested in 10 cohorts. For each cohort, behavioral testing was conducted across three days. Order of testing was novelty reactivity on day one, novelty preference on day two, and anxiety on day three. Littermates were tested on the same day using the same apparatus. Testing was conducted between 1000 and 1700. For all assays, mice were acclimated to the testing room for 10 min. Mice were picked up by the base of the tail to place them in the apparatus. Open field and novelty place preference testing were conducted in darkness. Elevated zero maze testing was conducted with illumination from a 15 W red light bulb.

#### Novelty reactivity

Mice were placed in the center of and facing the rear of the open field. The open field was covered to prevent mice from jumping out of the apparatus. The doors to the sound attenuating cubicle were closed during testing. Mice could explore the open field for 120 min after which they were removed and placed back in the home cage. Total distance traveled during testing was used as the index of novelty reactivity.

#### Novelty preference

This test consisted of an exposure phase and a testing phase. During the exposure phase, mice were placed in the center compartment of the apparatus with both doors closed. The door to the black or white side was opened (this variable was counterbalanced) and mice could explore for 15 min. Following this, mice were placed back in the center compartment with both doors closed. The testing phase immediately followed this. During the testing phase, both doors were open, and mice were allowed to explore both the familiar compartment (the one they explored during the exposure phase) and the novel compartment. Percentage of time in the novel compartment during the test phase was used as the index of novelty preference.

#### Anxiety

Testing procedures have been described in detail previously^19,20^. At the beginning of the test, mice were placed in and facing the center of the closed quadrant on the left half of the zero maze. Mice could explore the maze for five minutes after which they were removed and placed back in the home cage. Percentage of time in the closed quadrants was used as the index of anxiety.

### Statistical methods

Analysis of variance (ANOVA) was used to assess the effects of independent variables on behavioral endpoints. We performed factorial ANOVAs to examine the effects of housing condition, strain, and sex on novelty reactivity, novelty preference, and anxiety. Strain (C57BL/6J, DBA/2J) and sex were between-subjects factors in all ANOVAs. Housing condition (isolated, enriched) was a between-subjects factor in all ANOVAs except for those in which the difference between enriched and isolated littermates was used as the dependent variable. Normality of all measures was assessed by inspecting normal probability plots. The assumption of homogeneity of variance across groups and timepoints was assessed using Mauchly’s test of sphericity. The Huynh–Feldt correction was used when this assumption was violated. When performing multiple comparisons, Fisher's Least Significant Difference procedure was used. When analyzing novelty preference data, novel side (white/mesh, black/bars) was a between-subjects factor. Block (1–8) was a within-subjects factor when analyzing novelty reactivity data. Dependent variables for novelty reactivity, novelty preference, and anxiety were distance traveled in the open field, percentage of time spent in the novel compartment of the conditioned place preference apparatus, and percentage of time spent in the closed quadrants of the zero maze, respectively. To directly assess and visualize the effects of strain and sex on environmentally induced changes in novelty reactivity, novelty preference, and anxiety, we performed ANOVAs using the difference between enriched and isolated littermates on these behavioral phenotypes as the dependent variable. Table 1 shows experimental group sample sizes. Technical problems during testing required excluding eleven of these mice from the novelty preference analysis and five of these mice from the zero-maze analysis.

**Table 1:** Sample size of experimental groups.

Strain/sex	Housing condition	
	Isolated (*n* = 102)	Enriched (*n* = 102)
**C57BL/6J**		
Male	27	27
Female	25	25
**DBA/2J**		
Male	22	22
Female	28	28

Each isolation-housed mouse was matched to an enrichment-housed littermate that was housed on the same shelf side-by-side and tested in the same behavioral apparatus.

## Results

### Novelty reactivity, novelty preference, and anxiety were not significantly intercorrelated within experimental groups

We assessed phenotypic relationships within each of the experimental groups by calculating Pearson correlation coefficients (Table S1). Out of 40 tests, 3 of these correlations were significant at the 0.05 level. However, after adjusting for multiple tests using the Bonferroni correction, we observed no significant phenotypic relationships among novelty reactivity, novelty preference, and anxiety in any of the experimental groups. In addition to this, during exploratory analyses we performed the three primary ANOVAs presented in “Environmental enrichment reduced novelty reactivity in an open field significantly more in C57BL/6J mice relative to DBA/2J mice”, “Environmental enrichment reduced novelty preference in a three-chambered place-preference apparatus significantly more in DBA/2J mice relative to C57BL/6J mice”, “Environmental enrichment increased anxiety in an elevated zero-maze equivalently in C57BL/6J and DBA/2J mice” sections as ANCOVAs using the other two novelty/anxiety phenotypes as covariates. We found no significant effect of the covariates in these ANCOVAs.

### Environmental enrichment reduced novelty reactivity in an open field significantly more in C57BL/6J mice relative to DBA/2J mice

**Figure 1:**
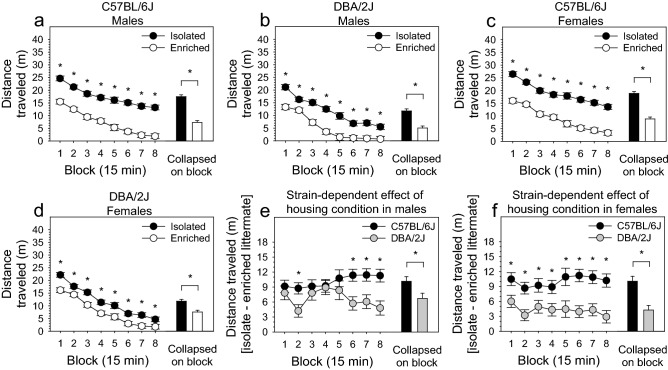
Environmental enrichment reduced novelty reactivity in an open field significantly more in C57BL/6J mice relative to DBA/2J mice. (**a**, **b**, **c**, **d)** Environmentally enriched mice explored the open field significantly less than isolated mice; this was true for both C57BL/6J and DBA/2J strains and for males and females within these strains (*p* < .05 for all tests). Males explored significantly less than females (*p* < .05) irrespective of housing condition and strain. DBA/2J mice explored significantly less than C57BL/6J mice in both the isolation and enrichment housing conditions (*p* < .05). (**e**, **f**) The reduction in novelty reactivity following environmental enrichment was significantly greater (*p* < .05) in the C57BL/6J strain than in the DBA/2J strain in both male and female mice.

Novelty reactivity was significantly influenced by housing condition [F (1, 196) = 220.83, *p* < 0.0001], strain [F (1, 196) = 60.06, *p* < 0.0001], sex [F (1, 196) = 6.71, *p* < 0.05], and the three-way interaction of housing condition, strain, and block [F (7, 1372) = 3.09, *p* < 0.05]. Post hoc tests indicated that, on all blocks, environmentally enriched mice explored the open field significantly less than isolated mice; this was true for both C57BL/6J and DBA/2J strains and for males and females within these strains (*p* < 0.05 for all tests) (Fig. 1a–d). Males explored significantly less than females (*p* < 0.05) irrespective of housing condition and strain. DBA/2J mice explored significantly less than C57BL/6J mice in both the isolation and enrichment housing conditions (*p* < 0.05); the magnitude of this strain difference was significantly greater in the isolation housing condition relative to the enrichment housing condition [housing × strain: F (1, 196) = 19.29, *p* < 0.0001].

To visualize the observed strain, sex, and block dependence of enrichment-induced reduction of novelty reactivity, we performed the same ANOVA as above with the following exception: we excluded housing condition as a factor and instead used the difference between enriched and isolated littermates as the dependent variable. Enrichment-induced reduction of novelty reactivity was significantly influenced by strain [F (1, 98) = 22.06, *p* < 0.0001] and the two-way interaction of strain and block [F (7, 686) = 3.13, *p* < 0.01]. Post hoc tests indicated that the reduction in novelty reactivity following environmental enrichment was significantly greater (*p* < 0.05) in the C57BL/6J strain than in the DBA/2J strain in both male and female mice (Fig. 1e,f).

### Environmental enrichment reduced novelty preference in a three-chambered place-preference apparatus significantly more in DBA/2J mice relative to C57BL/6J mice

**Figure 2:**
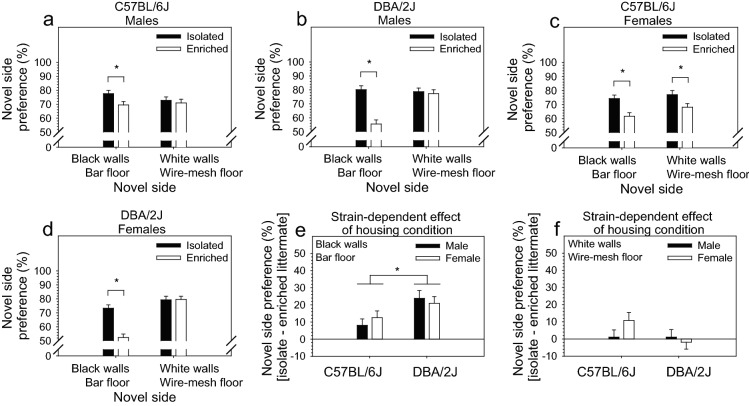
Environmental enrichment reduced novelty preference in a three-chambered place-preference apparatus significantly more in DBA/2J mice relative to C57BL/6J mice. (**a**, **b**, **c**, **d**) Environmentally enriched mice preferred the novel side significantly less than isolated mice; this relationship was true for both C57BL/6J and DBA/2J strains and for males and females within these strains (*p* < .05 for all comparisons). However, this relationship was strongly dependent on the characteristics of the novel side (i.e., white walls with a wire-mesh floor or black walls with a bar floor). Specifically, apart from C57BL/6J females, enrichment-induced reduction in novelty preference was exclusively observed when the novel side consisted of black walls with a bar floor. (**e**, **f**) Reduction in novel side preference following environmental enrichment was significantly greater in DBA/2J mice (*p* < .01), but only when the novel side consisted of black walls with a bar floor.

Novelty preference was significantly influenced by housing condition [F (1, 177) = 58.29, *p* < 0.0001], novel side [F (1, 177) = 33.43, *p* < 0.0001], the two-way interaction of sex and novel side [F (1, 177) = 6.08, *p* < . 05], and the three-way interaction of housing condition, strain, and novel side [F (1, 177) = 11.34, *p* < 0.01]. The three-way interaction of housing condition, strain, and sex approached significance [F (1, 177) = 2.75, *p* = 0.09]. Post hoc tests indicated that environmentally enriched mice preferred the novel side significantly less than isolated mice; this relationship was true for both C57BL/6J and DBA/2J strains and for males and females within these strains (*p* < 0.05 for all comparisons). However, this relationship was strongly dependent on the characteristics of the novel side (i.e., white walls with a wire-mesh floor or black walls with a bar floor) (Fig. 2a–d). Specifically, with the exception of C57BL/6J females, enrichment-induced reduction in novelty preference was exclusively observed when the novel side consisted of black walls with a bar floor.

To visualize the observed strain- and side-dependence of enrichment-induced reduction of novelty preference, we performed the same ANOVA as above with the following exception: we excluded housing condition as a factor and instead used the difference between enriched and isolated littermates as the dependent variable. Enrichment-induced reduction of novelty preference was significantly influenced by the two-way interaction of strain and novel side [F (1, 83) = 9.69, *p* < 0.01]. Post hoc tests indicated that reduction in novel side preference following environmental enrichment was significantly greater in DBA/2J mice (p < 0.01), but only when the novel side consisted of black walls with a bar floor (Fig. 2e,f).

### Environmental enrichment increased anxiety in an elevated zero-maze equivalently in C57BL/6J and DBA/2J mice

**Figure 3:**
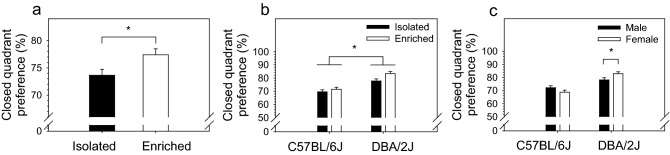
Environmental enrichment increased anxiety in an elevated zero-maze equivalently in C57BL/6J and DBA/2J mice. (**a**) Enriched mice spent significantly more time in the closed quadrants of the zero maze then isolated mice, irrespective of strain (*p* < .05). (**b**) DBA/2J mice spent significantly more time in the closed quadrants of the zero maze then C57BL/6J mice, irrespective of housing condition (*p* < .0001). (**c**) Female DBA/2J mice spent significantly more time in the closed quadrants of the zero maze then male DBA/2J mice (*p* < .05); time spent in the open quadrants of the zero maze by male and female C57BL/6J mice did not differ significantly.

Anxiety in a novel environment (i.e., time in the closed quadrants of a novel zero maze) was significantly influenced by housing condition [F (1, 191) = 5.93, *p* < 0.05], strain [F (1, 191) = 42.74, *p* < 0.0001], and the two-way interaction of strain and sex [F (1, 191) = 6.99, *p* < 0.01]. Post hoc tests indicated that enriched mice spent significantly more time in the closed quadrants of the zero maze then isolated mice, irrespective of strain (*p* < 0.05) (Fig. 3a). DBA/2J mice spent significantly more time in the closed quadrants of the zero maze then C57BL/6J mice, irrespective of housing condition (*p* < 0.0001) (Fig. 3b). Regarding the strain by sex interaction (Fig. 3c), female DBA/2J mice spent significantly more time in the closed quadrants of the zero maze then male DBA/2J mice (*p* < 0.05); time spent in the open quadrants of the zero maze by male and female C57BL/6J mice did not differ significantly.

## Discussion

In the present study, we assessed the effects of housing condition (environmental enrichment, isolation housing), mouse strain (C57BL/6J, DBA/2J), and sex on novelty reactivity, novelty preference, and anxiety. Littermates of the same sex were weaned at four weeks and housed in environmental enrichment or isolation for 10 weeks prior to testing. To control for variation in environmental variables that were not explicitly manipulated, each isolation-housed mouse was matched to an enrichment-housed littermate; these littermates were housed side-by-side on the same shelf throughout the experiment and tested in the same apparatus. Environmental enrichment significantly reduced exploration of a novel open field in males and females of both strains (Fig. 1a–d). This effect was significantly more robust in C57BL/6J mice relative to DBA/2J mice (Fig. 1e,f). Environmental enrichment significantly reduced preference for a novel chamber in a conditioned place preference apparatus in males and females of both strains; this effect was strongly dependent on chamber characteristics (i.e., wall color and floor material) (Fig. 2a–d) and was significantly more robust in DBA/2J mice relative to C57BL/6J mice (Fig. 2e,f). Environmental enrichment significantly increased anxiety in a zero-maze; this effect was not strain dependent in C57BL/6J and DBA/2J mice.

Importantly, the effect of strain on enrichment-induced behavioral change was completely dependent on phenotype: environmental enrichment conferred (1) a significantly greater effect on novelty reactivity in C57BL/6J mice, (2) a significantly greater effect on novelty preference in DBA/2J mice, and (3) an effect on anxiety that was statistically equivalent in C57BL/6J and DBA/2J mice. Collectively, these data indicate that environmental enrichment influences novelty reactivity, novelty preference, and anxiety via distinct genetic mechanisms in mice. Moreover, because the C57BL/6J and DBA/2J strains are the founders of the BXD recombinant inbred panel, these data indicate that the BXD panel can be used to identify the biological mechanism underlying these distinct gene x environment interactions.

### Environmental enrichment reduced novelty reactivity significantly more in C57BL/6J mice relative to DBA/2J mice

In the present study, environmental enrichment significantly reduced locomotion in a novel open field (i.e. novelty reactivity) in male and female C57BL/6J and DBA/2J mice (Fig. 1a–d), and this effect was significantly more robust in C57BL/6J mice (Fig. 1e,f). It is notable that the strain-dependent effect of environmental enrichment was robust throughout the entire session in females (Fig. 1f), whereas the effect in males (Fig. 1e) was most robust during the final 45 min of the 2-h session. This suggests that environmental enrichment interacts with distinct sex-specific genetic mechanisms to separately influence behavior during the early and late stages of the novelty reactivity assay (i.e., initial reactivity to a novel environment and habituation to a novel environment, respectively).

The attenuating effect of environmental enrichment on novelty reactivity has been widely observed in both rats and mice^12–15^. Indeed, this effect may be the most frequently observed behavioral consequence of home cage environmental manipulation. The influence of genetic background on this effect has been much less frequently investigated, but several studies in rats have found that strain significantly influences the attenuating effect of environmental enrichment on novelty reactivity^21–23^. In a study that is quite similar to the one reported here, Võikar and colleagues^24^ assessed novelty reactivity and other behavioral endpoints in mice from substrains of C57BL/6 and DBA/2 following either isolation housing or group housing (i.e., environmental enrichment limited to the social enrichment component). These authors reported that, as in the present study, isolation housed mice displayed significantly higher novelty reactivity relative to enriched mice. However, these authors did not observe a significant strain by environment interaction. Several methodological differences between the study reported here and the Võikar et al. study may explain this:

First, in the present study, we used multiple environmental enrichment components including access to conspecifics, exercise wheels, nesting materials, and toys. In contrast, the enrichment in Võikar et al. was limited to the presence of conspecifics. This suggests that social enrichment by itself is sufficient to attenuate novelty reactivity in these two mouse strains. Moreover, these findings suggest that (1) nonsocial and social components of environmental enrichment have distinct effects on novelty reactivity, and (2) nonsocial enrichment or the combination of social and nonsocial enrichment, but not social enrichment alone, interacts with strain to influence novelty reactivity in C57BL/6J and DBA/2J mice.

Second, in both the present study and the Võikar et al. study, mice were tested during the light component of the light:dark cycle. However, in the study reported here, novelty reactivity was quantified in darkness, whereas Võikar et al. quantified novelty reactivity in a well-illuminated cage (approximately 700 lux). These differences are relevant because at least two studies^23,25^ have found differences in novelty reactivity between enriched and isolated mice in dim or dark testing conditions but not lighted testing conditions. Importantly, one of these studies identified a housing × strain interaction on novelty reactivity under dim light but not bright light conditions^23^. This suggest that other phenomena, such as anxiety, may influence the expression of novelty reactivity in a strain- and environment-dependent manner.

Finally, the mice used in Võikar et al. were C57BL/6JOlaHsd and DBA/2OlaHsd which are distinct substrains from the C57BL/6J and DBA/2J mice used in the present study. Thus, genetic polymorphisms which have accumulated^26^ since the separation of these substrains may account for some of the behavioral differences observed in these two studies. Collectively, these data suggest that the strain dependent effect of environmental enrichment on novelty reactivity observed in C57BL/6J and DBA/2J mice in the present study was dependent on nonsocial aspects of environmental enrichment, qualities of the testing environment that influence phenotypes other than novelty reactivity (e.g., anxiety), the precise polymorphisms present in the JAX maintained substrains of C57BL/6 and DBA/2 mice, or some combination of these factors.

### Environmental enrichment reduced novelty preference significantly more in DBA/2J mice relative to C57BL/6J mice

In the present study, environmental enrichment significantly reduced preference for a novel chamber in a conditioned place preference apparatus (i.e. novelty preference) in male and female C57BL/6J and DBA/2J mice. In contrast to the effect of environmental enrichment on novelty reactivity, the effect of environmental enrichment on novelty preference has been infrequently studied. Studies that have investigated this phenomenon have found, as in the present study, that environmental enrichment attenuates novelty preference^25,27^.

A striking finding from the present study is that the effect of environmental enrichment on novelty preference was significantly more robust in DBA/2J mice relative to C57BL/6J mice (Fig. 2e,f). Notably, although environmental enrichment had the greatest effect on *novelty preference* in DBA/2J mice, it had the greatest effect on *novelty reactivity* in C57BL/6J mice. These distinct strain dependent effects suggest that novelty reactivity and novelty preference are driven by distinct genetic mechanisms; this hypothesis is consistent with previous research^8,28–30^. More importantly, these findings reveal that environmental enrichment influences these mechanisms differentially as a function of genetic background. This suggests that individuals are uniquely genetically vulnerable or resistant to the effects of environmental factors on novelty reactivity and, distinctly, on novelty preference.

The attenuating effect of environmental enrichment on novelty reactivity was not solely dependent on genetic background; it was also strongly dependent on chamber characteristics (i.e., wall color and floor material) (Fig. 2). Specifically, apart from female C57BL/6J mice, novelty preference was attenuated in environmentally enriched mice only when the novel side had black walls and floors made of bars. In contrast, when the novel side had white walls and floors made of wire mesh, the strong preference for the novel side exhibited by the isolation housed mice was equivalent to that exhibited by environmentally enriched mice (again, with the exception of C57BL/6J females). This effect may have been caused by preference for or aversion to specific characteristics associated with the two sides of the apparatus (i.e., side preference). Importantly, it is possible that the novelty preference phenotype and the side preference phenotype could vary independently as a function of strain, sex, housing condition, or an interaction among these factors. Moreover, these phenotypes could be apparatus specific. To address this potential confound, the side preference phenotype could be quantified in a separate apparatus-naïve cohort of mice by providing immediate access to both sides of the place preference apparatus. If a side preference exists, these data could be used as a covariate in the analysis of novelty preference. This would reduce effects unique to an apparatus and, consequently, enhance reproducibility and generalizability. However, as quantification of side preference and novelty preference both require the use of apparatus-naïve subjects, quantification of both phenotypes would effectively double the required sample size.

### Environmental enrichment increased anxiety equivalently in C57BL/6J and DBA/2J mice

In the present study, environmental enrichment significantly increased time in the closed quadrants of a zero-maze (anxiety). This effect was equivalent in C57BL/6J mice and DBA/2J mice (Fig. 3). Although environmental enrichment did not interact with strain to influence anxiety, sex interacted with strain such that DBA/2J females exhibited significantly elevated anxiety relative to DBA/2J males; male and female C57BL/6J mice did not differ in anxiety. These findings indicate that, in the tested strains and under the experimental conditions used in the present study, sex but not housing condition interacts with strain to influence anxiety.

Multiple studies have investigated the effects of environmental enrichment on anxiety using the elevated zero maze in mice^31–35^. Unlike the consistent findings that environmental enrichment attenuates novelty reactivity, the effects of environment enrichment on anxiety have been less clear. For example, zero maze studies using male mice, female mice, or both from C57BL/6 substrains have found increased anxiety (present study), decreased anxiety^31,34^, or no difference in anxiety^32,33^ relative to controls following varying types and degrees of environmental enrichment. The lack of clarity offered by these studies mirrors the variation in findings across the literature regarding the effects of environmental enrichment on anxiety^14,15^. Collectively, these studies suggest that variables including quality and duration of environmental enrichment, sex, testing apparatus, light intensity during testing, or a combination of these factors may influence anxiety and, consequently, the ability to detect strain dependent effects of environmental enrichment on anxiety.

It should be noted that some investigators add daily handling and weekly introduction of novel objects to their environmental enrichment protocol^36^. Neither of those strategies was used in this study. The addition of these enrichment components could reveal strain dependent effects of environmental enrichment on anxiety in C57BL/6J and DBA/2J mice that were not detected in this study. Moreover, the use of these enrichment components could enhance or diminish the strain dependent effects on novelty reactivity and novelty preference that were observed in this study.

### Environmental enrichment influences novelty reactivity, novelty preference, and anxiety via distinct genetic mechanisms in C57BL/6J and DBA/2J mice: relevance for discovery of the genetic and epigenetic mechanisms driving drug addiction.

In the present study, we directly compared the effects of environmental enrichment on three high-throughput, noninvasive phenotypes: novelty reactivity, novelty preference, and anxiety. We selected these phenotypes because each explains unique variance on the gold standard intravenous drug self-administration paradigm^3–8^. The most important finding from the present study is that environmental enrichment had a unique strain-dependent effect on each predictive phenotype: novelty reactivity was reduced significantly more in C57BL/6J mice, novelty preference was reduced significantly more in DBA/2J mice, and anxiety was significantly increased independent of strain. Collectively, this pattern of evidence reveals that environmental enrichment influences novelty reactivity, novelty preference, and anxiety via distinct genetic mechanisms in mice.

The findings presented here indicate that a systems genetics approach using the BXD recombinant inbred panel would enable discovery of the unknown genetic mechanisms driving the observed strain-dependent effects of environmental enrichment on intravenous drug self-administration^36,37^. To accomplish this, littermates from multiple BXD strains would be, as in the present study, housed in isolation or enrichment conditions. Using these mice, novelty phenotypes, anxiety phenotypes, and gene expression in addiction relevant brain regions would be quantified. Using these data, systems genetics analyses would enable identification of genetic loci associated with enrichment-induced variation in these endpoints. In the context of the housing manipulation used in the present study, genes identified using this approach would be strong candidates for validation on novelty phenotypes, anxiety phenotypes, and intravenous drug self-administration phenotypes. Moreover, BXD strains exhibiting extreme effects of environmental enrichment on multiple novelty and anxiety phenotypes could prove to be valuable models of environmentally induced addiction risk. The addition of ATAC-seq^38^ or MeDIP-seq^39^ to the systems genetics approach would enable discovery of underlying epigenetic mechanisms (i.e., chromatin accessibility, DNA methylation) driving the gene-by-environment interactions observed in the present study; in this regard, others have described epigenetic mechanisms underlying some effects of environmental enrichment^40,41^, the use of ATAC-seq in a systems genetics context^42^, and approaches for perturbation of the epigenome^43^. Collectively, these techniques offer a direct approach for discovering and validating genetic and epigenetic mechanisms through which environmental enrichment affects drug use and phenotypes which predict drug use.

## Supplementary Information

Supplementary Information
