# Supplementary material for: Environmental enrichment influences novelty reactivity, novelty preference, and anxiety via distinct genetic mechanisms in C57BL/6J and DBA/2J mice
Source: Sci Rep. 2021 Feb 16;11:3928. doi: 10.1038/s41598-021-83574-6 (PMC7887236; doi:10.1038/s41598-021-83574-6)
Supplement: Supplementary file 1 — Supplementary Information [file 41598_2021_83574_MOESM1_ESM.pdf]

Environmental enrichment influences novelty reactivity, novelty preference, and anxiety via distinct genetic mechanisms in C57BL/6J and DBA/2J mice

Price E. Dickson* and Guy Mittleman

Department of Psychology
University of Memphis
400 Innovation Drive
Memphis, TN 38111

* Corresponding author

Keywords:
addiction
substance use
reward
mouse
sensation seeking
novelty seeking
systems genetics
BXD

Current address of each author:

Price E. Dickson, Ph.D.
Department of Biomedical Sciences
Joan C. Edwards School of Medicine
Marshall University
1700 3rd Ave.
Huntington, WV 25703
price.dickson@marshall.edu

Guy Mittleman, Ph.D.
Department of Psychological Science
North Quad (NQ), room 104
Ball State University
Muncie, IN 47306
765-285-1960
gmittleman@bsu.edu

Table S1. Within-group correlation coefficients among the three novelty and anxiety phenotypes. Numbers in parentheses represent group sample size. Asterisks represent significance at the .05 level.

Experimental Group and Phenotype	Phenotype					
	Novelty reactivity		Novelty preference [white, mesh]		Novelty preference [black, bars]	
C57BL/6J, Isolated, Male						
Novelty preference [white, mesh]	.16	(13)	-		-	
Novelty preference [black, bars]	.33	(14)	-		-	
Anxiety	-.27	(25)	.01	(11)	-.22	(14)
C57BL/6J, Enriched, Male						
Novelty preference [white, mesh]	.20	(11)	-		-	
Novelty preference [black, bars]	.19	(14)	-		-	
Anxiety	-.06	(27)	-.44	(11)	.09	(14)
DBA/2J, Isolated, Male						
Novelty preference [white, mesh]	-.22	(12)	-		-	
Novelty preference [black, bars]	-.24	(10)	-		-	
Anxiety	-.26	(22)	.46	(12)	.02	(10)
DBA/2J, Enriched, Male						
Novelty preference [white, mesh]	-.08	(10)	-		-	
Novelty preference [black, bars]	.06	(9)	-		-	
Anxiety	.25	(21)	-.35	(9)	-.48	(9)
C57BL/6J, Isolated, Female						
Novelty preference [white, mesh]	-.25	(9)	-		-	
Novelty preference [black, bars]	.62*	(13)	-		-	
Anxiety	.12	(25)	.43	(9)	.06	(13)
C57BL/6J, Enriched, Female						
Novelty preference [white, mesh]	-.19	(12)	-		-	
Novelty preference [black, bars]	.12	(13)	-		-	
Anxiety	.32	(25)	-.39	(12)	-.19	(13)
DBA/2J, Isolated, Female						
Novelty preference [white, mesh]	-.15	(12)	-		-	
Novelty preference [black, bars]	-.27	(13)	-		-	
Anxiety	-.21	(27)	-.32	(11)	.62*	(13)
DBA/2J, Enriched, Female						
Novelty preference [white, mesh]	.09	(15)	-		-	
Novelty preference [black, bars]	.05	(13)	-		-	
Anxiety	-.39*	(27)	.30	(15)	.30	(12)
